# Cooperative Sensing Data Collection and Distribution with Packet Collision Avoidance in Mobile Long-Thin Networks

**DOI:** 10.3390/s18103588

**Published:** 2018-10-22

**Authors:** Lien-Wu Chen, Yu-Hao Peng, Yu-Chee Tseng, Ming-Fong Tsai

**Affiliations:** 1 Department of Information Engineering and Computer Science, Feng Chia University, Taichung 407, Taiwan; 2 Department of Computer Science, National Chiao Tung University, Hsinchu 300, Taiwan; yhpeng24@gmail.com (Y.-H.P.); yctseng@cs.nctu.edu.tw (Y.-C.T.); 3 Department of Electronic Engineering, National United University, Miaoli 360, Taiwan; mingfongtsai@gmail.com

**Keywords:** ad-hoc network, cycling group, fleet management, mobile long-thin network, sensor network

## Abstract

Mobile ad hoc networks (MANETs) have gained a lot of interests in research communities for the infrastructure-less self-organizing nature. A MANET with fleet cyclists using smartphones forms a two-tier mobile long-thin network (MLTN) along a common cycling route, where the high-tier network is composed of 3G/LTE interfaces and the low-tier network is composed of IEEE 802.11 interfaces. The low-tier network may consist of several path-like networks. This work investigates cooperative sensing data collection and distribution with packet collision avoidance in a two-tier MLTN. As numbers of cyclists upload their sensing data and download global fleet information frequently, serious bandwidth and latency problems may result if all members rely on their high-tier interfaces. We designed and analyzed a cooperative framework consisting of a distributed grouping mechanism, a group merging and splitting method, and a sensing data aggregation scheme. Through cooperation between the two tiers, the proposed framework outperforms existing works by significantly reducing the 3G/LTE data transmission and the number of 3G/LTE connections.

## 1. Introduction

The rapid technology developments of wireless communications and micro electro mechanical systems (MEMSs) have made *mobile ad hoc networks (MANETs)* possible. MANETs are infrastructure-less networks with mobile nodes, such as mobile devices, sensors, or vehicles. A MANET consisting of sensors forms a *mobile sensor network (MSN)* while one consisting of vehicles forms a *vehicular ad hoc network (VANET)*. A major challenge of MANETs is that no centralized coordination can be adopted. Mobile nodes in MANETs have to be self-organizing, and data collection and distribution among them have to be decentralized. In addition, with a single, shared communication channel, the well-known hidden and exposed terminal problems may raise in MANETs.

Several data collection and distribution mechanisms have been proposed for MANETs, which can be classified as *tree-based*, *mesh-based*, or *backbone-based* solutions. Tree-based solutions [1,2,3,4] use a tree to connect all multicast group members. However, links closer to the root suffer from heavier loads and failure of intermediate nodes is a serious issue. Mesh-based solutions [5,6,7] are more resilient to link failure, but the maintenance cost incurred is higher. Backbone-based solutions [8,9,10,11] use a backbone to avoid flooding the network when a new member joins a multicast group.

On the other hand, cycling has gained popularity in recent years due to concerns such as the environment and health [12]. Existing works include the bike-based mobile sensing system [13], sensor-based cyclist group tracking system [14], smartphone-based public bicycle sharing system [15], and networked bicycle platoon cooperation system [16]. In addition, multi-criteria urban bicycle routing [17] and cyclist crossing behavior model [18] are proposed for improving cycling experiences. A MANET with fleet cyclists using smartphones forms a two-tier *mobile long-thin network (MLTN)* along a common cycling route, where the high-tier network consists of 3G/LTE interfaces and the low-tier network consists of IEEE 802.11 interfaces.

In a fleet, individual sensing information (e.g., current position and activity, air quality, road vibration, etc.) and global status information (e.g., common messages, member locations, points of interests, social interactions, etc.) are frequently uploaded and downloaded by individual cyclists, respectively [19]. Although the energy-consuming problem using GPS for the up-to-date positions of cyclists is alleviated by cooperative energy-efficient localization [20], there are potential bandwidth and latency issues [21] that need to be addressed by optimizing sensing data collection and distribution among fleet cyclists. However, existing solutions for MANETs do not meet the requirements for a cycling fleet due to the following reasons: (1) The MANET topology is quite different from a cycling fleet. (2) The mobility patterns of a cycling fleet is not random; they follow a common cycling route. (3) The goal is to build a two-tier network while minimizing the high-tier cost.

Several clustering mechanisms [22,23,24,25,26,27,28] for sensing data collection and distribution have been proposed in vehicular ad hoc networks (VANETs). Clustering in VANETs is a process of grouping nearby vehicles within the communication range into a cluster according to various rules, such as the current speed, moving direction, final destination, etc. Clusters are virtual groups formed by a clustering mechanism. Each group has one or more group headers that are selected or elected by other group vehicles. Additional roles of vehicles, such as group relays, may be defined in some clustering mechanisms. Group size is different from one group to another, which depends on the communication ranges of onboard units used by vehicles. The vehicles in a one-hop group can communicate with each other via the group header or directly while those in a multi-hop group can transmit messages to all members through additional group relays. Since vehicles have constrained mobility patterns due to the road layout, they can be naturally grouped [29]. The structure of groups can be used as a virtual backbone consisting of the group headers [30], and specific data need to be collected and distributed in a given group [31].

However, most existing works focus on minimizing the low-tier message overhead instead of the high-tier communication cost. In addition, the mobility patterns of a fleet following a common route are not explored to efficiently collect and distribute sensing data [32]. The fleet management and fleet communication problems have to be considered for sensing data collection and distribution in two-tier MLTNs, where all fleet members need to connect to a backend server, transmit individual sensing information, and receive global status information.

Compared with the 3G/LTE network, the IEEE 802.11 network has high bandwidth with no charging fee [33]. In addition, the IEEE 802.11 channel can be reused by different ad-hoc groups in the same 3G/LTE base station. Furthermore, the broadcasting nature of the IEEE 802.11 network is efficient for transmitting global fleet information to multiple neighboring cyclists by only using a single broadcast message [34]. Therefore, a two-tier network is built to minimize the 3G/LTE cost.

To reduce the amount of 3G/LTE data transmitted and the number of 3G/LTE connections, we propose a cooperative framework for sensing data collection and distribution with packet collision avoidance in two-tier MLTNs to exploit ad-hoc low-tier communication as much as possible. Cyclists are divided into groups, and each group has a gateway to serve as the sensing data aggregator. Distributed grouping of cyclists is applied to maintain the network topology. Sensing data aggregation considers cyclists’ mobility patterns to minimize high-tier communication, which leads to an efficient solution for two-tier MLTNs. Simulation results show that the proposed framework outperforms existing works by significantly reducing the 3G/LTE data transmitted and the number of 3G/LTE connections.

The rest of this paper is organized as follows. Section 2 discusses existing works. Section 3 defines the problem of cooperative sensing data collection and distribution. Section 4 describes the proposed framework consisting of a distributed grouping mechanism, a group merging and splitting method, and a sensing data aggregation scheme. We analyze the high-tier communication cost in Section 5. Simulation results are presented in Section 6. Finally, Section 7 concludes the paper.

## 2. Related Work

Cycling is a green and healthy style for short-distance movement, which has been studied in [13,14,15,16]. Eisenman et al. [13] designed and implemented a mobile networked sensing system, called BikeNet, for cyclists to provide the healthiness of a given route, such as pollution levels, allergen levels, noise levels, and roughness of the terrain. BikeNet is a delay tolerant solution that allows cyclists to take cycling trips, collect sensed data, and upload their data as wireless access points exist. Shin et al. [14] designed a sensor-based tracking system for cyclist groups to provide each bicycle with a green power source, establish the riding path without a GPS device, and monitor cyclist trajectories and group positions. Zhao et al. [15] developed a smartphone-based public bicycle sharing system for cyclists to query the numbers of bicycles in rental spots and predict the available number of returning bicycles. In addition, cyclists can estimate their calorie expenditure during a riding trip and keep tracking their physical exercises. Cespedes et al. [16] investigated cyclist platoon cooperation for traveling at a necessary speed to arrive at the destination in a given time and maintaining a proper distance with the bicycle in front.

On the other hand, a VANET is a distributed network of vehicles that can collect and distribute safety and traffic information on roads. There are various clustering mechanisms [22,23,24,25,26,27,28] proposed for sensing data collection and distribution in VANETs. Table 1 compares the features of VANET clustering schemes and ours in terms of grouping principle, optimization goal, group size, network interface, and simulation tool. Taleb et al. [22] designed a risk-aware media access control (MAC) protocol to associate an emergency level with each vehicle in its corresponding cluster for increasing the responsiveness of the collision avoidance system. Vehicles are grouped based on their speeds, moving directions, and inter-vehicle distances. In a cluster, each vehicle is assigned an emergency level to reflect the risk associated with that particular vehicle to fall into a collision with other cars. The proposed risk-aware strategy delivers warning messages to the drivers with the shortest latencies, which could either prevent chain collisions or reduce the associated damage.

Benslimane et al. [23] addressed clustering-based gateway management including gateway selection, advertisement, and discovery in a VANET-3G integrated network. Clustering is performed based on the moving direction of vehicles, received signal strength of 3G, and inter-vehicle distance for link stabilities. The minimum number of gateways is selected to connect cluster vehicles with the 3G network, and even some vehicles without 3G interfaces can still access the 3G network through VANET communications to gateways. Thus, frequent handoffs at 3G base stations and the associated signaling overhead can be avoided by preventing all vehicles from connecting directly to the 3G base station. Nevertheless, the mobility patterns of a fleet following a common route are not considered in the clustering and gateway management.

Chen et al. [24,25] dynamically formed warning groups on the road to prevent rear-end collisions through vehicle-to-vehicle communications. A warning group is formed by a sequence of vehicles in the same lane such that each vehicle does not keep a safety distance from the vehicle in its front except the first one. When any vehicle of a group takes an emergency brake, a warning message will be sent immediately to those vehicles behind the braking vehicle in the same group. The warning messages may be delivered through multi-hop forwarding with the highest priority. Thus, drivers can become aware of such emergency braking event even before they actually see the braking signals.

Remy et al. [26] used the Long Term Evolution (LTE) network as the fixed infrastructure to centrally group vehicles into single-hop clusters within at most the communication range of IEEE 802.11p, whereas our proposed framework can group vehicles into multi-hop clusters in a distributed manner. In [26], the base station tries to create clusters with a lifetime as long as possible. In each cluster, a cluster header is elected, which has the responsibility to send its floating car data (e.g., position, speed, and direction) and its cluster members to the base station via LTE. Cluster members send their floating car data only via 802.11p to the cluster header that aggregates the floating data of cluster members before sending the floating data to the base station. In particular, the cluster head can avoid sending the useless data of a vehicle with the unchanged heading and velocity to the base station. Thus, LTE bandwidth usage can be reduced by alleviating redundant data transmissions. In addition, a compression algorithm can be used on the aggregated data to further save LTE bandwidth.

Rawashdeh et al. [27] employed the dedicated short range communications (DSRC) based on the IEEE 802.11p PHY and MAC layers to enable vehicle-to-vehicle communications. DSRC utilizes 75 MHz bandwidth from 5.850 to 5.925 GHz that is divided into seven channels, where one of the channels is the control channel for safety applications and the remaining six ones are service channels for non-safety applications. The speed difference between neighboring vehicles is considered for constructing a stable clustering structure. Stable and unstable clustering neighbors are defined according to their speeds and relative moving directions. Clusters are formed only among stable neighbors such that vehicles show similar mobility patterns in one cluster. Thus, fast moving vehicles are grouped on the fast speed lanes in one cluster while slow moving vehicles are grouped in another cluster, which can increase the cluster lifetime and reduce the transition of vehicles between clusters.

Zhou et al. [28] analyzed the event-driven warning message propagation process (i.e., detecting an accident) to suggest a suitable message lifetime for guaranteeing the all relevant vehicles (in the zone of relevance) can be notified before reaching the potential danger. The analytical models are proposed to investigate the propagation process in a connected network (where all relevant nodes are in the same group) and a partitioned network (where the relevant nodes are in different groups). In addition, the delivery success probability of warning messages is derived for different traffic conditions. Moreover, a direction-aware broadcast protocol is proposed to reduce warning message redundancy during multi-hop broadcast in the partitioned network.

## 3. System Model

**Figure 1:**
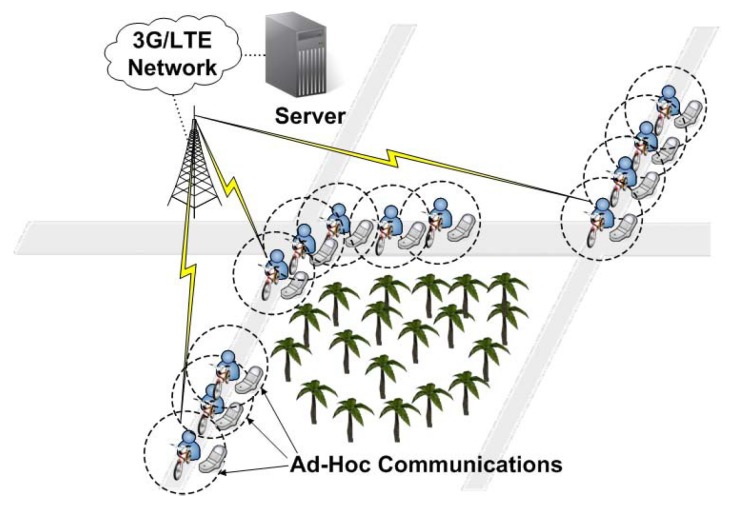
A two-tier mobile long-thin network formed by fleet cyclists.

Figure 1 shows the system architecture of two-tier MLTNs and an example of fleet cyclists forming three ad-hoc (low-tier) communication groups. We consider cyclists in a fleet moving along a common cycling route. All cyclists use smartphones with both 3G/LTE and IEEE 802.11 interfaces, which form the high-tier network and the low-tier (ad-hoc) network, respectively. A backend server manages sensing data exchange. Current individual sensing and global fleet information are frequently uploaded to the server and downloaded from the server by cyclists, respectively. The high-tier network is assumed to be always connected. However, the use of the high-tier network has to be limited to save bandwidth and reduce latency. Therefore, the use of the low-tier network has to be maximized to encourage local communication. To facilitate sensing data collection, the cyclists are divided into different groups, where each has a gateway node. Cyclists in the same group can communicate with each other via the low-tier network. Only the gateways transmit collected sensing data to the backend server via the high-tier network. Sensing data distribution occurs via the gateways, which use the high-tier network to receive sensing data from the server and the low-tier network to forward sensing data to members.

An undirected graph G=(V,E) is used to represent the low-tier network, where *V* is the set of all fleet members and *E* is the set of all links. Because the cyclists have a common cycling path, *G* is divided into a number of long-thin groups that can be sorted in order with its low-tier links. During the sensing data collection process, the members of each group transmit their sensing data to the gateway using the low-tier network. The gateway then transmits the collected sensing data to the server via the high-tier network. During the sensing data distribution process, the server sends global fleet information to all gateways (via the high-tier network), and they in turn broadcast the received fleet information to group members (via the low-tier network). Due to the different speeds of cyclists, a group could split separately and two groups could merge together. The following issues are addressed to minimize the high-tier communication cost in two-tier MLTNs:

1. *Cyclist Grouping*: How should cyclist groups be managed as cyclists join or leave, to ensure that the gateway can receive the sensing data of all members and transmit it to the server?
2. *Group Merging and Splitting*: How should groups be merged and split according to cycling speeds so that an existing gateway can be removed and a new gateway can be selected, respectively?
3. *Transmission Reordering*: How should the transmission order of members for sensing data collection be determined with the aim of minimizing packet collision probabilities in the low-tier network?
4. *Data Aggregating*: How should the sensing data of each member be aggregated to minimize the member sensing data uploaded and global fleet information downloaded via the high-tier network?

## 4. The Proposed Framework

In this section, we present the proposed framework for grouping cyclists into clusters in a distributed manner (in Section 4.1), merging one group with another group, splitting one group into two different groups (in Section 4.2), aggregating member sensing data as much as possible, and updating the transmission order of all members (in Section 4.3).

### 4.1. Distributed Grouping of Cyclists

All fleet members in *G* are supposed to ride along a known common path *P*. In a group, a cyclist may act as a *Group Header (GH)*, a *Relay Cyclist*, or an *Ordinary Cyclist*. The first cyclist of a group will act as the GH to broadcast beacons whereas other group members may act as relay cyclists to forward beacons or simply as ordinary cyclists. Within a group, each cyclist is assigned a serial number (SN), where the SN of the first cyclist (i.e., the GH) is 1, the SN of the second cyclist is 2, …, and the SN of the *i*th cyclist is *i*. The SN of the last cyclist in a group is equal to the current group size. Each GH periodically broadcasts *Invitation Advertisement Beacons (IABs)*, where an IAB contains the fleet ID, group ID, sender ID, sender position, sender SN (SN${}_{s}$), current group size, maximum group size, and sequence number. The fleet ID and group ID convey whether the IAB is from the same fleet and the same group, respectively, because there may be different fleets cycling on the same path and each fleet consists of one or more groups. Based on the sender ID and position, an IAB receiver can calculate the distance between the sender and itself. Thus, it can select a location-based backoff timer such that the receivers further from the same sender could rebroadcast earlier on receiving IABs. SN${}_{s}$ is the SN of the GH or relay cyclist that sent the IAB. The purpose of IABs is to broadcast the existence of a group and to invite nearby fleet cyclists as new members. A cyclist can join a group only if the current group size is smaller than the maximum group size that is depending on the allowed round-trip delay for cooperative sensing data collection and distribution.

**Figure 2:**
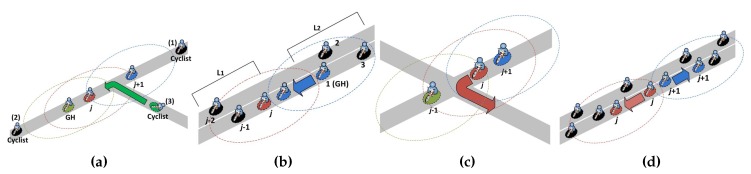
(**a**) A new fleet cyclist joins an existing group *L*; (**b**) groups L${}_{1}$ and L${}_{2}$ are merged; (**c**) group cyclist *j* changes its direction and thus group *L* is split; and (**d**) group cyclist *j* speeds up and/or group cyclist j+1 slows down, thus group *L* is split.

When a fleet cyclist *c* turns on a smartphone, it listens for IABs from its fleet for θ seconds. If no IAB is received, it will form a new group and act as its GH. Otherwise, depending on the content of the received IAB, there are three possible cases for cyclist *c* to join an existing group *L* as follows, where the current group size is *n* (i.e., there are *n* members in the group currently), as shown in Figure 2a. Note that if multiple IABs of *L* are received, cyclist *c* joins *L* based on the IAB with the smallest SN${}_{s}$.

1. If SN${}_{s}$ = *n*, the IAB was sent by the last group cyclist in *L*. Cyclist *c* is currently behind the last group cyclist, it can join *L* from the rear. Cyclist *c* will set its SN to n+1 and broadcast a join message $M_{join}$ to all group cyclists ahead to update the current group size to n+1. Otherwise, cyclist *c* can join *L* from the front and middle according to Case 2 and Case 3, respectively.
2. If SN${}_{s}$ = 1, the IAB was sent by the GH in *L*. Cyclist *c* is in the front of the GH, it can join *L* from the front. Cyclist *c* will set the group ID to its ID, the current group size to n+1, and its SN to 1 to act as the new group header GH${}_{new}$ and send a notification message $M_{notify}$ to the old group header GH${}_{old}$ to replace it. Then, GH${}_{old}$ will set its SN to 2 and broadcast an update message $M_{update}$ to all group cyclists *i* following behind, to set the group ID to the ID of GH${}_{new}$, the current group size to n+1, and SN${}_{i}$ to SN${}_{i}+1$. Otherwise, cyclist *c* can join *L* from somewhere in the middle according to Case 3.
3. If 1 < SN${}_{s}$<*n*, the IAB was sent by a relay cyclist in *L* and cyclist *c* can join *L* somewhere in the middle. Cyclist *c* will send a request message $M_{req}$ with its GPS location to one-hop neighboring group cyclists for requiring the two closest group cyclists, group cyclists *j* and j+1, to reply with ACK${}_{j}$ and ACK${}_{j+1}$. After receiving ACK${}_{j}$ and ACK${}_{j+1}$, cyclist *c* will set its SN to SN${}_{j}+1$ and broadcast a $M_{join}$ message to all group cyclists ahead to update the current group size to n+1. In addition, cyclist *c* will broadcast a $M_{update}$ message to all group cyclists *i* behind it to update the current group size to n+1 and SN${}_{i}$ to SN${}_{i}+1$.

In particular, those $M_{join}$, $M_{notify}$, $M_{update}$, and $M_{req}$ messages also contain the IAB content that is received by the joining fleet cyclist *c*. If multiple fleet cyclists join *L* at the same time, then the GH can use the contained IAB contents to determine the correct group size. For example, when two fleet cyclists *c* and $c^{\prime }$ broadcast different $M_{join}$ messages to update the current group size to n+1 concurrently, the GH can detect this concurrent joining of cyclists *c* and $c^{\prime }$ and then update the current group size to n+2 if these two $M_{join}$ messages contain the same IAB content. In addition, the incorrect SNs (if any) of group cyclists due to the concurrent joining (or IAB message lost) can be further corrected by the transmission reordering process proposed in Section 4.3. Note that due to the errors of GPS localization, the geographical positions of the first (last) group cyclist with SN =1 (SN =n) may not be in the front (back) of all other group cyclists. However, this only causes our system to exchange extra messages, but would not cause problems.

### 4.2. Merging and Splitting of Groups

Next, we discuss how a group can merge with another group and be split into two different groups. As shown in Figure 2b, if the group cyclists of a group $L_{1}$ of size $n_{1}$ that is ahead reduce their speeds or the group cyclists of a group $L_{2}$ of size $n_{2}$ that is behind increase their speeds, the last group cyclist of $L_{1}$ and the first group cyclist (i.e., GH${}_{2}$) of $L_{2}$ may become neighboring cyclists that can communicate with each other. If the GH${}_{2}$ of $L_{2}$ receives the IAB${}_{1}$ of $L_{1}$, GH${}_{2}$ will broadcast a $M_{notify}$ message to all group cyclists in $L_{1}$ to update the current group size to $n_{1}+n_{2}$. In addition, GH${}_{2}$ will broadcast a $M_{update}$ message to all group cyclists *i* in $L_{2}$ to update the group ID to the ID of GH${}_{1}$, the current group size to $n_{1}+n_{2}$, and SN${}_{i}$ to SN${}_{i}+n_{1}$. Thus, all group cyclists of $L_{2}$ join $L_{1}$ (i.e., $L_{2}$ is merged into $L_{1}$). Note that if the total number of group cyclists in $L_{1}$ and $L_{2}$ are larger than the maximum group size, the group cyclist with the SN equal to the maximum group size plus one will form a new group to meet the requirement for the allowed round-trip delay of cooperative sensing data collection and distribution.

On the other hand, when one or more group cyclists leave from a group, this group may be split into two smaller groups. If the group does not split after the group cyclists have left, the serial numbers of the remaining group cyclists can be corrected by the reordering process described in Section 4.3. Otherwise, a group *L* of size *n* can split in the following ways, as shown in Figure 2c,d.

1. As shown in Figure 2c, when group cyclist *j* changes its direction, group cyclists j-1 and j+1 cannot communicate with each other and thus *L* is split. At this time, if group cyclist j-1 cannot receive the rebroadcasted IAB from the back after relaying an IAB, it becomes the last group cyclist in *L* and broadcast a $M_{notify}$ message to all group cyclists ahead to update the current group size to SN${}_{j-1}$. On the other hand, if group cyclist j+1 cannot receive any IAB from *L* within θ seconds, it will form a new group and act as its GH. Then, group cyclist j+1 will broadcast a $M_{update}$ message to all behind group cyclists *i* to update the group ID to its ID, the current group size to n-SN${}_{j}$, and SN${}_{i}$ to SN${}_{i}-$SN${}_{j}$.
2. As shown in Figure 2d, after either group cyclist *j* speeds up or group cyclist j+1 slows down, group cyclists *j* and j+1 cannot communicate with each other, thus *L* is split. Similarly, if group cyclist *j* cannot receive the rebroadcasted IAB from the back after relaying an IAB, it will broadcast a $M_{notify}$ message to all group cyclists ahead to update the current group size to SN${}_{j}$. On the other hand, if group cyclist j+1 does not receive any IAB from *L* within θ seconds, it will form a new group and act as its GH. Then, group cyclist j+1 will broadcast a $M_{update}$ message to all behind group cyclists *i* to update the group ID to its ID, the current group size to n-SN${}_{j}$, and SN${}_{i}$ to SN${}_{i}-$SN${}_{j}$.

In particular, if *L* splits into three or more groups when multiple group cyclists leave simultaneously, the group sizes and serial numbers of group cyclists in these splitting groups can be updated by the reordering process proposed in Section 4.3.

### 4.3. Sensing Data Aggregation in Groups

Finally, we discuss how global fleet information can be cooperatively collected and distributed in two-tier MLTNs consisting of cyclist groups. To improve the sensing data collection of group cyclists, the GH is selected as the gateway in a group. During sensing data collection for a group of size *n*, group cyclist *i* will transmit its sensing data $SD_{i}$ to group cyclist i-1 in sequence, according to the order SN${}_{i}=n,n-1,\cdots ,1$. Thus, after group cyclist *i* receives the sensing data $SD_{n},SD_{n-1},\cdots ,$ and $SD_{i+1}$ from group cyclist i+1, it can immediately send the received sensing data and $SD_{i}$ to group cyclist i-1 without collisions with the sensing data of group cyclists n,n-1,⋯,i+1 because all group cyclists follow a common route and transmit their sensing data in order. In addition, group cyclist i-1 will aggregate the sensing data of group cyclist *i* based on the relative position instead of the absolute position. Thus, the GH (i.e., group cyclist 1) receives the aggregated sensing data of all group cyclists from group cyclist 2 via the low-tier network and transmits it to the server via the high-tier network.

On the other hand, the sensing data distribution from the GH to group cyclist *n* occurs as follows. After the server has collected global fleet information from all groups in *G*, it transmits global fleet information to all GHs in *G*. Then, each GH broadcasts global fleet information to the group cyclists behind it through multi-hop forwarding. When group cyclist *n* receives global fleet information, it will transmit its sensing data to group cyclist n-1 again, thus repeating the process of cooperative sensing data collection and distribution. Note that the transmission order of sensing data collection is one by one from cyclist *n* to cyclist 1 (i.e., the GH) following their serial numbers so that there is no message contention during the sensing data collection process and the packet collision probability within a group can be minimized.

Because each group cyclist may increase or decrease its speed, its SN in the group may change with time. To obtain the correct SN${}_{i}$ of all group cyclists *i*, before the GH transmits the aggregated sensing data to the server, it will broadcast a *Reordering Beacon (ROB)* to all group cyclists to update their serial numbers. The ROB contains a current serial number SN${}_{c}$ during the reordering process, which is equal to 1 when the GH broadcasts it. The receiver closest to the GH will rebroadcast first as it receives the ROB. When group cyclist *i* receives a ROB, it will calculate the distance $d_{i}$ between the sender and itself. A smaller value of $d_{i}$ is used to set a smaller backoff timer as follow:

(1) $$\begin{array}{c} BT_{i}^{ROB}=\left\{\begin{array}{cc} \left[0,2^{\tau +1}-1\right] & 0<d_{i}\leq \frac{1}{\rho }r\\ \left[2^{\tau +1},2^{\tau +2}-1\right] & \frac{1}{\rho }r<d_{i}\leq \frac{2}{\rho }r\\ \;\vdots \\ \left[2^{\tau +\rho -1},2^{\tau +\rho }-1\right] & \frac{\rho -1}{\rho }r<d_{i}\leq r \end{array}\right., \end{array}$$

where ρ is the number of backoff classes, *r* is the transmission range, and τ is a small integer. When the group cyclist closest to the sender backs off to 0, it will set its SN to SN${}_{c}+1$ and rebroadcast the ROB with SN${}_{c}=$ SN${}_{c}+1$. The reordering process will be repeated until the last cyclist in the group rebroadcasts the ROB and then broadcasts a $M_{update}$ message to all group cyclists ahead to update the current group size to its SN. Thus, all cyclists in the group will have the up-to-date group size and serial numbers.

To reduce the number of rebroadcasts of IABs, $M_{join}$, $M_{update}$, $M_{notify}$, $M_{req}$, and global fleet information, the receivers that are further from the sender will rebroadcast earlier as they receive IABs, $M_{join}$, $M_{update}$, $M_{notify}$, $M_{req}$, and global fleet information. Below, we use IABs as an example. When group cyclist *i* receives an IAB, *i* will calculate the distance $d_{i}$ between the sender and itself. Different from ROBs in the reordering process, a larger value of $d_{i}$ will set a smaller backoff timer $BT_{i}$ for IABs, as defined below:

(2) $$\begin{array}{c} BT_{i}=\left\{\begin{array}{cc} \left[0,2^{\tau +1}-1\right] & \frac{\rho -1}{\rho }r<d_{i}\leq r\\ \left[2^{\tau +1},2^{\tau +2}-1\right] & \frac{\rho -2}{\rho }r<d_{i}\leq \frac{\rho -1}{\rho }r\\ \;\vdots \\ \left[2^{\tau +\rho -1},2^{\tau +\rho }-1\right] & 0<d_{i}\leq \frac{1}{\rho }r \end{array}\right., \end{array}$$

where ρ, *r*, and τ are the same with the parameters used in $BT_{i}^{ROB}$. Thus, receivers further from the sender have higher priorities to rebroadcast the IAB [24,25]. For example, there are receivers *A*, *B*, and *C* whose distances from the sender are 250 m, 150 m, and 50 m, respectively. When r=300, τ=1, and ρ=3, receivers *A*, *B*, and *C* will randomly select their backoff timers $BT_{A}$, $BT_{B}$, and $BT_{C}$ from [0,3], [4,7], and [8,15], respectively, because $200<d_{A}\leq 300$, $100<d_{B}\leq 200$, and $0<d_{C}\leq 100$. Thus, the farthest receiver (i.e., cyclist *A*) can backoff to 0 earlier and immediately rebroadcast the IAB received from the sender because $BT_{A}<BT_{B}<BT_{C}$.

In addition, an implicit acknowledgement (ACK) strategy is adopted to eliminate redundant IABs. Specifically, the reception of an IAB from the relay cyclist behind group cyclist *i* serves as an implicit ACK that prevents group cyclist *i* from competing again. On receiving such a rebroadcast, group cyclist *i* will remove the IAB in its waiting queue. In the rebroadcasted IAB, the sender ID, position and SN will be replaced by the ID, position and SN of the relay cyclist. Furthermore, to improve reliability, a relay cyclist that already broadcast the IAB will try to detect any rebroadcast from the group cyclists behind it. If no such rebroadcast is detected, it will send a rebroadcast again with a new sequence number. Under the scheme of distance-based backoff timers, such rebroadcasting will occur no more than once. If the relay cyclist cannot detect any rebroadcasting after it rebroadcasts the IAB again, it will become the last group cyclist and check whether the current group size is equal to its SN. If not, it will broadcast a $M_{update}$ message to all group cyclists ahead to update the current group size to its SN.

## 5. Analysis of 3G/LTE Cost

For analyzing 3G/LTE cost, a cycling fleet *F* consisting of one or more long-thin groups is considered. We describe the assumptions (which are similar to [25,35]) used in our analytical model and derive the expected amounts of cyclist sensing data $d_{L}$ uploaded (i.e., $d_{E}^{\mathrm{UL}}$) and global fleet information $d_{F}$ downloaded (i.e., $d_{E}^{\mathrm{DL}}$) via 3G/LTE in a group *L*. We use a Poisson distribution to approximately model the number of fleet cyclists in any subinterval of the common cycling path *P* and thus the distance between any two consecutive fleet cyclists is exponentially distributed. Each fleet cyclist rides on *P* with a constant speed and has a fixed transmission range of *r*. If the distance between two fleet cyclists is equal to or less than *r*, they can directly communicate with each other.

Suppose that the total number of fleet cyclists on *P* of length *C* is *N* and the fleet cyclists on *P* are $b_{1},b_{2},\ldots ,$ and $b_{N}$. The size of *L* is decided by two conditions. The first condition is the density λ(=N/C) of fleet cyclists on *P*. The second condition is the distance $d_{i,i+1}$, for i=1,2,…,N-1, of consecutive fleet cyclists $b_{i}$ and $b_{i+1}$. In particular, $d_{E}^{\mathrm{UL}}$ and $d_{E}^{\mathrm{DL}}$ may increase as λ increases or $d_{i,i+1}$ decreases. A random variable $D_{i}$ that represents the distance between $b_{i}$ and $b_{i+1}$ (for i=1,2,…,N-1) is exponentially distributed with the following cumulative distribution function:

(3) $$F_{D_{i}}\left(d\right)=\left\{\begin{array}{cc} 1-e^{-\lambda d} & d\geq 0\\ 0 & d<0 \end{array}\right..$$

Assume that *L* consists of *n* group cyclists (i.e., exactly n-1 consecutive pairs of fleet cyclists are within *r*). It is required that $D_{i}\leq r$ for i=1,2,…,n-1 and $D_{n}>r$ if n<N, whereas it is required that $D_{i}\leq r$ for i=1,2,…,n-1 if n=N. The probability $P_{n}$ that has *n* group cyclists in *L* follows:

(4) $$P_{n}=\left\{\begin{array}{cc} \prod_{i=1}^{n-1}Pr\left\{D_{i}\leq r\right\}\times Pr\left\{D_{n}>r\right\} & n<N\\ \prod_{i=1}^{n-1}Pr\left\{D_{i}\leq r\right\} & n=N \end{array}\right..$$

$D_{1},D_{2},\ldots ,D_{n-1},$ and $D_{n}$ are independent and identically distributed random variables. From Equations (3) and (4), by substituting $1-e^{-\lambda r}$ and $e^{-\lambda r}$ for $Pr\{D_{i}\leq r\}$ and $Pr\{D_{n}>r\}$, respectively, $P_{n}$ follows:

(5) $$P_{n}=\left\{\begin{array}{cc} {\left(1-e^{-\lambda r}\right)}^{n-1}e^{-\lambda r} & n<N\\ {\left(1-e^{-\lambda r}\right)}^{n-1} & n=N \end{array}\right..$$

Therefore, the expected number $n_{GC}$ of group cyclists in *L* is

(6) $$n_{GC}=\sum_{i=1}^{N}P_{i}\times i.$$

The expected number $n_{L}$ of groups in *F* is

(7) $$n_{L}=\frac{N}{n_{GC}}.$$

In our framework, each group cyclist *j* transmits its $d_{L}$ to group cyclist j-1 in sequence via ad-hoc communication and then the GH (i.e., group cyclist 1) transmits the aggregated $d_{L}$ to the server via 3G/LTE. Let $d_{H}$ and $d_{P}$ represent the sizes of the packet header and absolute position of $d_{L}$, respectively. Let α be the sensing data aggregation ratio using relative positions instead of absolute positions. The expected $d_{E}^{\mathrm{UL}}$ of $d_{L}$ uploaded via 3G/LTE for sensing data collection can be modeled by summing the expected size of aggregated $d_{L}$ over all possible numbers of group cyclists in *L* as follow:

(8) $$d_{E}^{\mathrm{UL}}=\sum_{i=1}^{N}P_{i}\times \left[d_{H}+d_{P}+\alpha \times \left(i-1\right)\times d_{P}\right].$$

After collecting all $d_{L}$ in *F*, the server returns $d_{F}$ to the GH. The expected amount $d_{E}^{\mathrm{DL}}$ of $d_{F}$ downloaded via 3G/LTE for sensing data distribution can be modeled by multiplying the expected number of groups in *F* and the expected amount of the aggregated $d_{L}$ uploaded in *L* as follow:

(9) $$d_{E}^{\mathrm{DL}}=n_{L}\times d_{E}^{\mathrm{UL}}.$$

On the other hand, in the single-tier scheme (without ad-hoc communication), every group cyclist in *L* has to report its $d_{L}$ to the server for sensing data collection and then obtains $d_{F}$ from the server for sensing data distribution. The expected amount $d_{E}^{\mathrm{UL}}$ of $d_{L}$ uploaded via 3G/LTE in *L* is equal to $n_{GC}\times \left(d_{H}+d_{P}\right)$. As there are *N* absolute positions of cyclists in *F*, the size of $d_{F}$ is $d_{H}+N\times d_{P}$ and thus the expected amount $d_{E}^{\mathrm{DL}}$ of $d_{F}$ downloaded via 3G/LTE in *L* is equal to $n_{GC}\times \left(d_{H}+N\times d_{P}\right)$.

**Figure 3:**
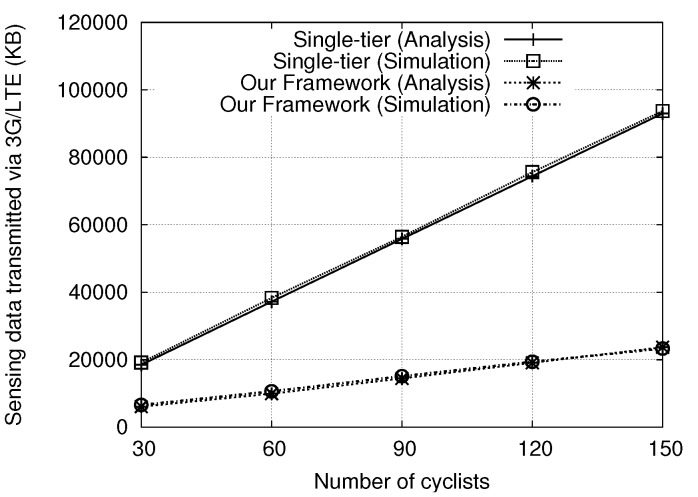
Comparisons of simulation and analytical results.

Figure 3 shows the comparison of analytical and simulation results for the single-tier scheme and our proposed framework. We verify the derived amount of sensing data transmitted via 3G/LTE through simulations with a Poisson distribution of cyclists, where the simulation environment and other parameters are described in Section 6. Clearly, the simulated and analytical results are quite close to each other, which justifies the correctness of our derivation.

## 6. Performance Evaluation

The single-tier scheme, LTE4V2X [26], and the proposed framework were implemented in the Qualnet 5.0 [36] simulator to compare their performance in terms of the sensing data transmitted via 3G/LTE and the number of 3G/LTE connections. For fairness, we only cumulated the amount of cyclist sensing data uploaded and global fleet information downloaded by the group headers in LTE4V2X. The total number of cyclists in the fleet network varied from 30 to 150. We randomly placed all cyclists on a common cycling path and randomly set their moving speeds from 20 to 40 km/h every 100 m. The network interface of IEEE 802.11b with two-ray ground radio model was adopted and its maximum transmission range was 283 m in Qualnet (for open spaces with line of sight). The ratio of aggregated and raw data sizes for a cyclist’s sensing information was 0.25. Each cyclist updated her/his sensing data every 5 s and each gateway broadcasted an IAB every 5 s. θ was set to 5 and the simulation time was 900 s. Each simulation was repeated 100 times and the average value was taken. Simulation parameters are summarized in Table 2.

**Figure 4:**
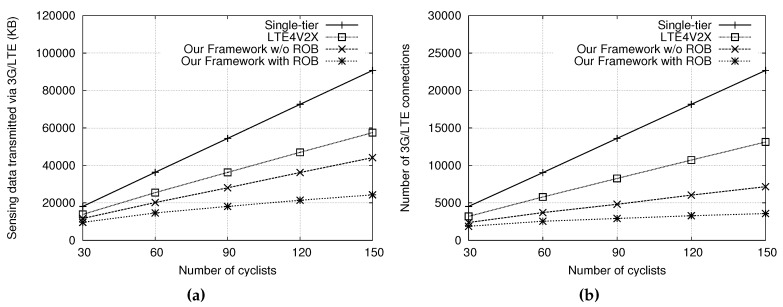
Comparisons of: (**a**) the sensing data amount transmitted via 3G/LTE; and (**b**) the numbers of 3G/LTE connections under the sensing data update interval of five seconds.

Figure 4a illustrates the sensing data transmitted via 3G/LTE under different numbers of cyclists. The total numbers of cyclists were set to 30, 60, 90, 120, and 150. In Figure 4a, it can be observed that our scheme transmitted many fewer sensing data via 3G/LTE; only one-fourth and one-third of the sensing data transmitted of the single-tier scheme and LTE4V2X with 150 cyclists, respectively. This is because our scheme can locally collect member sensing data and only the gateway has to transmit the aggregated sensing data to the server via 3G/LTE. In addition, global fleet information is only downloaded by the gateway via 3G/LTE and then broadcast to group members via multi-hop forwarding in the low-tier network. In contrast, the single-tier scheme requires all cyclists to transmit their sensing data to the server and receive fleet information from the server via 3G/LTE, and LTE4V2X has more gateways to transmit sensing data without data aggregation and to download fleet information than our approach. Therefore, both schemes have to transmit many sensing data via 3G/LTE with numbers of cyclists.

Figure 4b shows the number of 3G/LTE connections made for different numbers of cyclists in the fleet network, where many TCP connections result in a long round-trip time of packets [37]. As the number of cyclists increases, the numbers of 3G/LTE connections for the single-tier scheme and LTE4V2X increase exponentially, but the number of 3G/LTE connections for our scheme only increases linearly. The single-tier scheme and LTE4V2X require establishing larger numbers of 3G/LTE connections than our scheme because all cyclists and numbers of gateways have to create connections to the server, respectively. In our scheme, few gateways have to make connections to the server, thus the number of 3G/LTE connections is minimized. Note that the impact of road topology is small on the proposed framework because the fleet cyclists ride along the same cycling path and have the same turning direction at intersections or T-way roads. In addition, the signal fading and multi-path effects of cycling routes in rural and suburban areas are less than those of driving routes in urban areas with high buildings [38,39].

**Figure 5:**
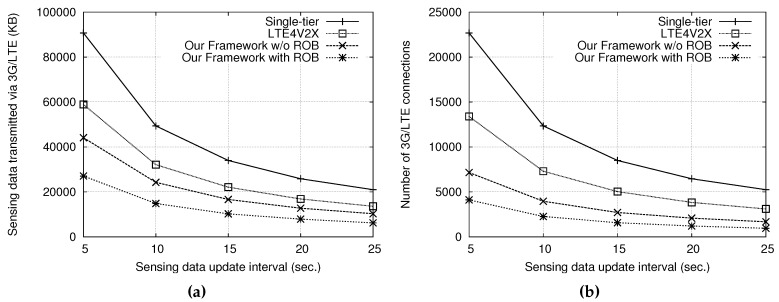
Comparisons of: (**a**) the sensing data amount transmitted via 3G/LTE; and (**b**) the numbers of 3G/LTE connections under different sensing data update intervals.

In addition, Figure 4a,b shows the performance improvement achieved by reordering the transmission order of cyclists. The sensing data transmitted via 3G/LTE and the number of 3G/LTE connections established were further reduced after reordering. This is because a group cyclist following an incorrect transmission order may create a new group if it is located in the front of its group header, thus increasing the number of gateways. On the other hand, Figure 5a,b shows the sensing data transmitted via 3G/LTE and the numbers of 3G/LTE connections with 150 cyclists for different sensing data update intervals, respectively, where both the IAB interval and θ are equal to the sensing data update interval (i.e., 5 s). The results show that the 3G/LTE data transmitted and the number of connections can be reduced by setting a larger interval for sensing data updates, but, in that case, the sensing data are less current. In particular, our approach can maintain up-to-date sensing information for cyclists while keeping the 3G/LTE cost low.

## 7. Conclusions

This paper studies cooperative sensing data collection and distribution with packet collision avoidance in two-tier MLTNs consisting of 3G/LTE high-tier interfaces and IEEE 802.11-based low-tier interfaces. In the proposed framework, we designed a distributed grouping mechanism, a group merging and splitting method, and a sensing data aggregation scheme. Our framework can significantly reduce the amount of sensing data transmitted via 3G/LTE and the number of 3G/LTE connections, which leads to more efficient utilization of 3G/LTE bandwidth. The adoption of our scheme in two-tier MLTNs can solve the scalability problem resulting from limited 3G/LTE bandwidth, and prevent fleet cyclists from getting disconnected due to long 3G/LTE delays. The framework allows the efficient exchange of sensing data and status information among fleet cyclists. 

## Figures and Tables

**Table 1:** Comparison of existing clustering mechanisms [22,23,24,25,26,27,28] and our framework.

Features	Grouping Principle	Optimization Goal	Group Size	Network Interface	Simulation
reference [22]	inter-vehicle distance	low-tier delivery latency	multi-hop	DSRC (802.11p)	NS-2
reference [23]	link stability	high-tier signaling overhead	multi-hop	3G + DSRC (802.11p)	NS-2
reference [24,25]	safety distance	low-tier clustering overhead	multi-hop	DSRC (802.11p)	Qualnet/C++
reference [26]	cluster lifetime	high-tier bandwidth usage	one-hop	LTE + DSRC (802.11p)	NS-3
reference [27]	speed difference	low-tier stable structure	one-hop	DSRC (802.11p)	C++
reference [28]	zone of relevance	low-tier message lifetime	multi-hop	DSRC (802.11p)	NS-2
our framework	fleet member	high-tier bandwidth usage	multi-hop	3G/LTE + Wi-Fi (802.11a/b/g)	Qualnet

**Table 2:** Simulation parameters.

Parameter	Value
Simulation time	900 s
Number of cyclists	30∼150
Cycling speed	20∼40 km/h
Network interface	IEEE 802.11b
Path loss model	Two Ray (n = 2)
Frequency band	2.4 GHz
Channel bandwidth	20 MHz
Carrier sensing threshold	−88 dBm
Transmission range	283 m
Rate adaptation mechanism	Auto Rate Fallback (ARF)
Data aggregation ratio	0.25
Data update interval	5∼25 s
Number of simulation runs	100
